# PAIN, PAIN MANAGEMENT, AND CONSEQUENCES OF PAIN AMONG HOSPITALIZED PERSONS WITH DEMENTIA

**DOI:** 10.1093/geroni/igz038.1672

**Published:** 2019-11-08

**Authors:** Rachel Arendacs, Marie Boltz, Ashley Kuzmik, Barbara Resnick

**Affiliations:** 1 Penn State University College of Nursing, State College, Pennsylvania, United States; 2 Pennsylvania State University, University Park, Pennsylvania, United States; 3 Penn State College of Nursing, State College, Pennsylvania, United States; 4 University of Maryland School of Nursing, Baltimore, Maryland, United States

## Abstract

The purpose of this study was to describe the incidence, management and impact of pain on behavior and delirium in hospitalized older adults living with dementia. This was a descriptive study using baseline data from patients in the first cohort of the Fam-FFC study which evaluates the impact of a family-engaged function-focused care intervention in hospitalized patients with dementia. The majority of the sample was female (70%) and black (80%) with a mean age of 82.5 (SD=8.9). Pain (PAINAD) was reported by 36% of the patients; 42% of those demonstrating pain were prescribed analgesics. Controlling for age, gender and baseline cognition, pain was significantly associated with behavioral and psychiatric symptoms of dementia ( t =2.1, p= .034) and delirium severity (t = 4.9, p<.0001). Results suggest the need for pain assessment and individualized treatment plans to promote comfort and decrease behavioral and delirium symptoms in hospitalized persons with dementia.

